# Performance Evaluation of Fluorescent Polymer Gel Microspheres as a Reservoir Conformance Control Agent

**DOI:** 10.3390/gels11020085

**Published:** 2025-01-22

**Authors:** Saya Shagymgereyeva, Bauyrzhan Sarsenbekuly, Wanli Kang, Sarsenbek Turtabayev

**Affiliations:** 1 School of Energy and Petroleum Industry, Kazakh-British Technical University, Almaty 050000, Kazakhstan; 2 Faculty of Natural Science, Zhanibekov University, Shymkent 160012, Kazakhstan

**Keywords:** polymer gel microspheres, fluorescent polymer microspheres, oil recovery, particle size, thermal stability

## Abstract

This study introduces fluorescent polymer gel microspheres (FPMs) as a novel approach to enhance conformance control in oil reservoirs. Designed to address the challenges of high-permeability zones, FPMs were synthesized via inverse suspension polymerization, incorporating 2-acrylamido-2-methylpropane sulfonic acid (AMPS) to improve thermal stability and swelling and fluorescein to enable fluorescence. Characterization using FT-IR, SEM, fluorescence spectroscopy, and thermal analysis revealed that FPMs swell significantly in brine, with diameters increasing from 46 μm to 210 μm, and maintain thermal stability up to 110 °C. These advanced properties make FPMs highly effective in reducing permeability and facilitating real-time tracking, offering a promising solution for improved oil recovery and efficient reservoir management.

## 1. Introduction

Oil reservoirs generally have a significant number of fractures or high permeability channels named “thief zones” or “streaks”, which are caused by long-term water flooding. Subsequently, thief zones lead to low efficiency of water flooding, excessive water production, and poor production rate in the final phases of field development [1]. Nowadays, the petroleum industry is experiencing a lack of advanced methods and conformance control technologies. Gels have been introduced as a solution to block water flow, redirecting it from highly permeable areas to zones with lower permeability [2,3,4]. Polymer microspheres have emerged as a new trend in gel treatments due to their ability to overcome inherent limitations in in situ gelation systems. These microspheres serve as viscoelastic agents with a tridimensional structure, which are able to assimilate formation water and flow through the porous reservoir medium under injection pressure. According to the tests completed in the Shengli, Dagang, and Jidong oil fields in China, the potential of polymer microspheres as effective conformance control agents, particularly in heterogeneous and fractured reservoirs, was proven [5,6,7]. Studies by Hua and Lin, among others, delved into the characteristics, rheological properties, plugging capabilities, profile control mechanisms, and oil displacement mechanisms of these nanoscale polymer microspheres [8]. Their findings revealed that these microspheres can reduce water permeability by adsorbing, accumulating, and bridging in pore throats. Under pressure, the adsorbed layers collapse, enabling the microspheres to penetrate deeply into the reservoir, facilitated by their excellent deformation properties.

Research by Yao, Wang, Yang, Kang, Yang, Xie, and their teams explored various aspects related to polymer microspheres [9,10,11,12,13,14,15]. These investigations covered the effects of ionic strength on the transport and retention of polyacrylamide microspheres in porous media, mechanisms influencing the initial particle size and swelling capabilities of polymer microspheres under synthesis and reservoir conditions, as well as the optimization of injection parameters for polymer microspheres and polymer composite flooding systems [11]. Studies conducted in the Shengli, Dagang, and Jidong oil fields have demonstrated the efficacy of polymer microspheres as a conformance control tool in mature reservoirs. Research by Hua et al. has highlighted that these microspheres can significantly reduce water permeability by absorbing excess water. This absorption leads to the decomposition of adsorbed layers under high pressure, which depicts dual-labile and non-labile structured cross-linker polymer microspheres [16,17,18].

Recent advancements have revealed that relying solely on quantitative assessments of plugging behavior and compliance mechanisms falls short of delivering a comprehensive understanding of reservoir dynamics. To bridge these gaps, fluorescent polymer microspheres have been proposed. These microspheres serve dual purposes: they function as conformance control agents that help align fluid flow within the reservoir and also serve as tracers to indicate the presence and distribution of oil deposits. Their application in oil field supervision marks a significant advancement in optimizing oil recovery operations [19].

Fluorescent polymer microspheres (FPMs)—specially engineered microspheres that incorporate fluorescent properties to allow for real-time tracking in reservoir applications display numerous chemical and physical properties that are successfully utilized in multiple fields owing to their unique composition. The integration of Flu within the FPM matrix imparts fluorescent ability to the polymer [20]. This feature facilitates its use in fluorescence-based applications in the form of a tracer agent in conformance control systems. 2-Acrylamido-2-methylpropane Sulfonic Acid (AMPS)—a monomer known for enhancing the swelling and thermal stability of polymers, which is critical in high-temperature environments, Acrylamide (AM)—a monomer used for its hydrophilic properties in polymer synthesis, N-Vinylpyrrolidone (NVP)—a monomer that enhances the hydrophilicity and thermal resistance of polymers. Fluorescein (Flu)—a fluorescent dye that allows the polymer to be tracked in real time under UV light. The inclusion of AMPS and AM in the copolymer structure endows FPM with hydrophilic properties, enabling it to absorb water and swell [21,22]. These characteristics are advantageous for applications involving water retention or controlled release systems. The sulfonic acid groups from AMPS provide ion-sensitive behavior, making FPM responsive to changes in the surrounding ionic environment. This property renders FPM potentially suitable for ion-responsive materials used in sensing or selective ion adsorption applications [23]. The combination of AM, AMPS, and Flu permits the tuning of the polymer’s properties, including mechanical strength. The modifications that FPMs underwent increased the range of applications of the polymer. Its responsiveness to ions and capacity of fluorescence emission serve as a solution for environmentally conscious monitoring, water treatment, and analytical methods that necessitate ion detection or tracking capabilities. Nowadays, FPMs are progressively implemented in profile control strategies, where fluid flow paths in reservoirs can be effectively controlled, and the oil production rate is significantly improved. The range of specific applications for FPM can be further customized through various synthesis modifications and targeted functionalities, thereby meeting the specialized needs of distinct conditions of different oil reservoirs [24].

Introducing rigid rings, large side groups, or inflexible components in molecular design can significantly enhance polymer temperature resistance [23,24,25]. The monomer AMPS contains large side groups such as sulfonate (–SO_3_H) and dimethyl [–C(–CH_3_)_2_], effectively boosting the steric resistance of the monomers and thereby improving the temperature resilience of resulting polymers. Moreover, the strong polar nature of SO_3_H contributes excellent hydrophilic properties, which not only enhance temperature resistance but also improve the water absorption characteristics of the resultant microspheres [26,27]. This study investigates a certain kind of FPM aimed to become a novel conformance control agent. The microspheres were covalently dyed with Flu by inverse suspension polymerization [28,29]. Analysis of the FPM was performed by fluorescence microscopy, environmental scanning electron microscopy (ESEM), and scanning electron microscopy (SEM), a high-resolution imaging technique used to observe the surface morphology of microspheres, providing detailed structural information [29,30,31,32,33,34]. The research covered the structure, morphology, swelling behavior, and thermal stability of the FPM, underlining the significance of the microspheres’ prolonged thermal stability within a microsphere/water dispersion system.

## 2. Results and Discussion

### 2.1. Structure Characterization of the FPM

**Figure 1:**
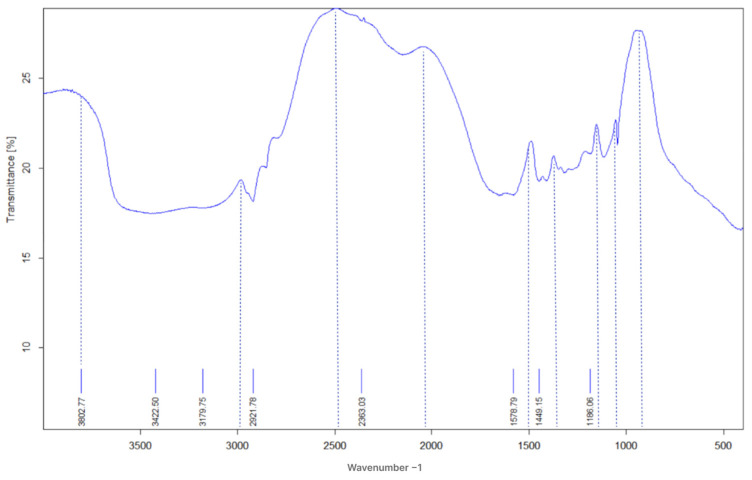
The FTIR spectrum of terpolymer microspheres.

Figure 1 illustrates the FT-IR spectrum of the terpolymer microspheres, indicating key functional groups present in the polymer structure. This structural characterization is crucial for understanding how these functional groups contribute to the swelling and thermal properties of the FPM [16]. Notably, a distinct double-peak observed in the AM spectrum, ranging from 3100 to 3800 cm^−1^, the appearance of the N–H stretching vibration peak at 3000 cm^−1^ indicates the presence of acrylamide units within the polymer microsphere FPM. Additionally, the C=O bending vibration absorption peaks 15,000–1700 cm^−1^, suggesting partial hydrolysis of the amide to the carboxyl group within the polymer microspheres. Peaks between 1000–1300 cm^−1^, possibly around 1000–1100 cm^−1^ for S=O stretching, and around 1200–1300 cm^−1^ for S–O stretching observed in AMPS. Another discernible peak under 1000 cm^−1^ is attributed to the bending vibration of aromatic hydrogen due to the minimal presence of Fluorescein in the side chains that exhibit reduced intensity in the spectra. The presence of these specific peaks in the FT-IR spectrum confirms the successful incorporation of both acrylamide and AMPS into the polymer structure [35,36,37,38,39,40,41]. Moreover, the observed cross-linked network indicates that the FPMs are well-engineered for enhanced performance in challenging reservoir conditions. This intricate structure not only contributes to their swelling capabilities but also enhances their mechanical stability, making them suitable for effective conformance control in oil recovery processes.

**Figure 2:**
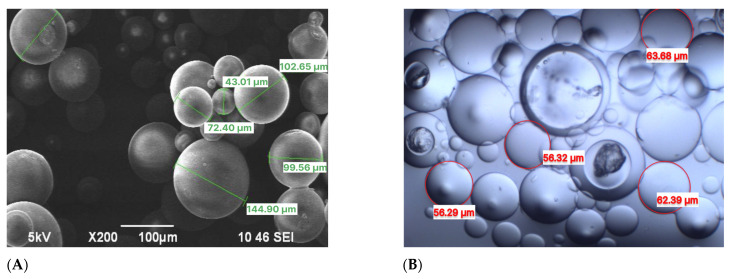
Particle size of FPMs: (**A**) SEM images, (**B**) optical microscope images.

Figure 2 demonstrates particle size analysis and reveals the tridimensional cross-linked networks of FPM before and after swelling at 25 °C. The synthesized FPMs exhibit gel-like properties, characterized by their ability to swell significantly and maintain structural integrity under varying conditions.

Upon contact with brine water, these FPMs, through the influence of internal and external osmotic pressure, allow water molecules to penetrate the internal network. This action stretches the molecular chains within the network structure, thereby increasing the volume of the polymer microspheres [42]. The average particle sizes of the FPMs before and after swelling are measured at 46 μm and 215.6 μm, respectively.

### 2.2. Fluorescent Property of FPM

**Figure 3:**
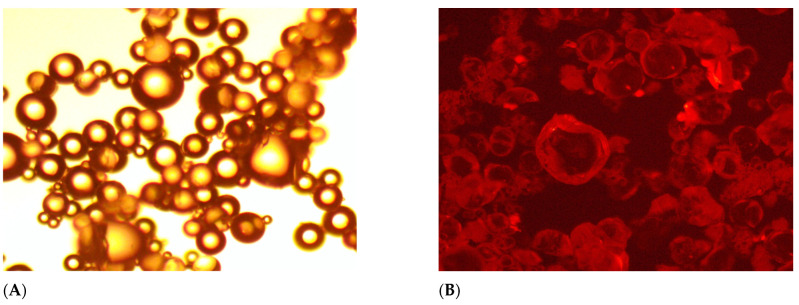
Morphology of FPMs: (**A**) under the ordinary light, (**B**) under the UV light.

The fluorescent behavior of the FPM is examined here, with figures demonstrating fluorescence under UV light. This characteristic allows for real-time tracking and monitoring of FPM within the reservoir [43,44]. Moreover, observations from Figure 3A,B reveal that the swollen polymer microspheres FPM exhibit fluorescent properties. Under UV light, these polymer microspheres emit distinct fluorescence, while under regular light, they appear as colorless and transparent spheres.

The occurrence of fluorescence capacity can be attributed to the increased conjugation effect of the fluorescent monomer fluorescein upon being grafted onto the polymer microspheres [37,45,46,47,48,49,50]. This effect led to a reduction in the energy associated with electron transition, subsequently resulting in the lengthening of the fluorescence emission wavelength. The following results of analysis of the FPM’s swelling behavior and thermal stability demonstrate the effectiveness of our FPMs as a conformance control agent under challenging reservoir conditions [51].

### 2.3. Swelling Property of FPM

Table 1 summarizes the swelling behavior of various polymer microspheres, detailing their initial sizes and measurements after 8, 24, and 72 h of exposure to brine. The data show that the FPM demonstrated significant swelling capability, increasing from 46.62 μm to 210.52 μm, which is critical for their effectiveness in conformance control. This information underscores the FPM’s potential for enhanced oil recovery applications by illustrating their ability to adapt in high-salinity environments. Due to the addition of AMPS, the diameters of the microspheres decreased while their swelling capacity increased. After 8 h of swelling, we observed a sharp increase in diameter. The growth continued for up to 24 h. However, after 72 h, the microspheres’ diameter reached a stable level, suggesting that they had reached their swelling limit by the 24-h mark. Beyond this time, no further increase in diameter was detected. The swelling behavior observed in the FPM is indicative of gel formation, allowing the microspheres to effectively encapsulate and retain water, which is critical for conformance control. An analysis of the swelling behaviors of FPM and AM-NVP-Flu, both of which contain fluorescein, suggests that fluorescein alone does not have a significant effect on the swelling properties of these polymer microspheres. Despite the addition of fluorescein, the FPM displayed a high swelling capacity, while AM-NVP-Flu exhibited comparatively low swelling. This discrepancy points to other factors as primary determinants of swelling behavior, specifically the cross-linking density and the presence of hydrophilic groups within the polymer matrix [48].

**Figure 4:**
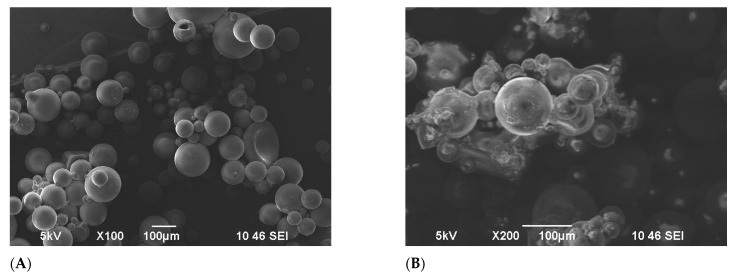
SEM images of terpolymer microspheres: (**A**) before swelling, (**B**) after swelling.

Figure 4 shows the penetration of brine water into the matrix of the FPM; the concentration of the brine used was 29.5 g/L NaCl. Figure 4A exhibits an SEM image displaying the terpolymer microspheres in a dry-powdered state, showing initial particle diameters of 46 μm on average. In Figure 4B, another SEM image showcases the microspheres post-24 h of swelling. At this stage, the sphere diameters ranged between 100–200 μm. Despite the swelling, the microspheres retained their spherical shape while experiencing a significant increase in size. To deepen the understanding of the swelling behavior of the FPM, it is essential to recognize that the interaction between the polymer network and the brine solution is primarily governed by osmotic pressure differentials. Additionally, the presence of functional groups within the polymer matrix facilitates water absorption through hydrogen bonding, thereby enhancing the swelling capacity. The tridimensional cross-linked architecture of the microspheres allows for substantial deformation while maintaining structural integrity, a critical characteristic for their application in diverse fluid environments.

These microspheres, characterized by a tridimensional cross-linked structure and numerous free hydrophilic groups (–CONH_2_) within, readily bond with polar brine water molecules through hydrogen bonding. The novelty of this study lies in the finding that the incorporation of fluorescent functional groups into the polymer microspheres does not significantly impact their swelling properties despite the presence of these groups. This insight is crucial as it demonstrates that the enhanced swelling observed is primarily due to the intrinsic polymer structure and hydrophilic content rather than the fluorescent additives [40,42,43,44]. Particle size distribution analysis is essential for understanding the size range of polymer microspheres, as it must align with the pore throat dimensions in reservoirs to optimize swelling and fluid absorption. A narrow and controlled size distribution is preferred, as uniform microspheres facilitate consistent swelling behavior and enhance their ability to block and divert fluids. Conversely, a broad distribution may lead to uneven swelling, negatively impacting the overall effectiveness of enhanced oil recovery (EOR) processes.

Swelling capacity testing evaluates the microspheres’ ability to absorb fluids without compromising their structural integrity, which is vital for maintaining performance during injection and in situ conditions. High swelling capacity is crucial to ensure that microspheres can effectively expand and interact with reservoir fluids; inadequate swelling may reduce their efficacy and overall sweep efficiency.

Adsorption performance assesses the microspheres’ ability to interact with specific components in the reservoir, such as oil and water. Understanding their adsorption behavior is critical for evaluating their selective swelling and blocking properties, allowing for customization to meet the specific needs of the reservoir environment.

In summary, particle size distribution, swelling capacity, and adsorption performance are fundamental parameters for the design and evaluation of polymer microspheres in EOR applications. An optimized combination of these properties enhances conformance control, improves fluid interaction, and facilitates effective blocking and diversion, ultimately contributing to increased oil recovery.

### 2.4. Temperature Resistance of Fluorescent Microspheres FPM

**Figure 5:**
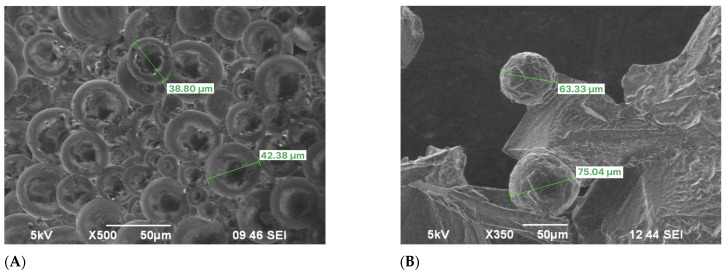
SEM images of terpolymer microspheres: (**A**) the 8th day, (**B**) the 14th day 110 °C temperature exposure.

The FPM underwent an observation in an oven at a concentration of 5000 mg/L and 110 °C. The progression of the dispersion system was monitored over several days, revealing notable changes. Initially, the microspheres significantly expanded upon swelling, and this swelling continued to intensify over time [47,48]. A distinct boundary between the solid and liquid phases became visible. By the 8th day, the microspheres exhibited noticeable swelling, as shown in Figure 5A. Over the following days, the swelling continued, and by the 14th day at 110 °C, the surface of the microspheres began to lose its smoothness, displaying slight irregularities without any signs of evident degradation, as depicted in Figure 5B.

**Figure 6:**
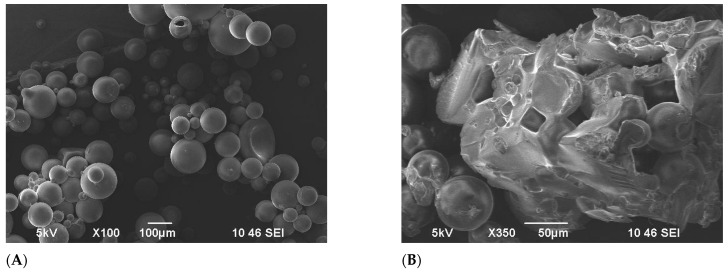
SEM images of terpolymer microspheres: (**A**) before temperature exposure, (**B**) the 15th day 110 °C temperature exposure.

However, after this period, the particle size of the microspheres began to diminish, gradually losing their three-dimensional structure. Figure 6B shows the SEM pictures of FPM by the 15th day of 110 °C exposure, where the deformation of microspheres is clearly observed. By the 19th day, all the microspheres had completely degraded.

**Figure 7:**
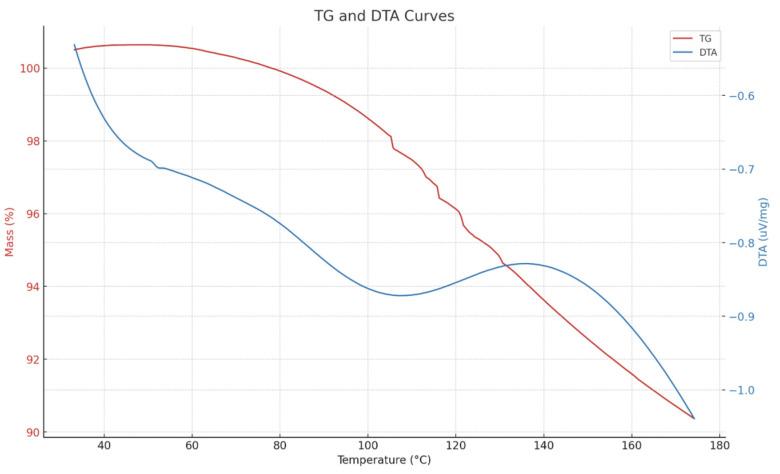
The TG curves of FPM.

The thermal gravimetric analysis (TGA) curve of the polymeric microspheres is depicted in Figure 7. It is evident from Figure 7 that the thermogravimetric behavior of the polymeric microspheres can be categorized into three stages. Initially, in the first stage spanning from 25 °C to 104 °C, the 5% TGA temperature range of the polymeric spheres is observed, attributed to the evaporation of adsorbed water on their surface. Subsequently, in the second stage spanning from 104 to 300 °C, with an initial decomposition temperature of 104 °C, the fracture and decomposition of amido and carboxyl groups within the polymer molecular chain of the microspheres occur. The latter range commences from 300 to 600 °C, where 300 °C is the breakdown point: the tridimensional structure of FPM loses its integral shape. On the basis of TGA data assessment, the polymeric microspheres possess excellent thermal stability until they reach their breaking point. The thermal deformation takes place in three obvious stages, representing the continuous degradation of different particles in the polymer matrix. The TG/DTA analysis revealed that the FPMs exhibit exceptional thermal stability and act as advanced gel systems suitable for challenging reservoir environments. This enhanced temperature resilience represents a novel breakthrough, making FPMs particularly suitable for challenging reservoir conditions. These results highlight the potential of FPMs to improve oil recovery processes in conditions where conventional microspheres might not be effective.

The temperature resistance of polymers used in Enhanced Oil Recovery (EOR) applications depends on the specific polymer type, reservoir conditions, and EOR objectives. Typically, polymers such as polyacrylamide and partially hydrolyzed polyacrylamide are employed to modify fluid properties and enhance oil recovery, generally functioning effectively within a temperature range of approximately 10 °C (50 °F) to 80 °C (176 °F). While many EOR polymers perform well at lower temperatures, only a limited subset, including high-temperature polyacrylamides, are designed for high-temperature reservoirs, capable of withstanding temperatures up to 120 °C or higher.

In EOR planning, the careful selection, testing, and monitoring of polymers are essential to ensure they can endure the reservoir’s thermal conditions while effectively improving sweep efficiency and oil recovery rates. The FPM demonstrates considerable thermal resilience, attributed to its three-dimensional structure. When subjected to temperatures ranging from 25 °C to 110 °C, these microspheres exhibit notable swelling properties. Their resilience in harsh reservoir environments, combined with good elasticity and selective plugging performance, positions them as advantageous materials. Flooding experiments conducted in porous media have indicated that these microspheres maintain their integrity without fracturing under stress [8].

However, it is imperative to conduct comprehensive investigations into the plugging capabilities of fluorescent microspheres to further enhance conformance control technologies. Elevated temperatures can adversely impact the stability of low-elastic microspheres, initiating thermal decomposition beyond 60 °C. As the temperature increases, the volume viscosity decreases, facilitating the immersion of low-elastic microspheres and compromising the structural integrity of the entire network. Therefore, accurately assessing the effects of formation temperature on the system’s solidity is critical when designing oil displacement processes. Ensuring the stability of the oil displacement mechanism is vital for effectively treating deeper reservoir layers and achieving targeted profile control objectives [25].

The thermal stability of FPM was assessed, and the results indicate that FPMs exhibit a notably high resistance to thermal degradation, particularly in temperature ranges exceeding 100 °C. This enhanced stability is essential for applications in challenging reservoir environments where high temperatures can compromise the efficacy of conventional polymer agents. The superior thermal stability of FPMs can be attributed to the presence of AMPS in its structure. AMPS introduces sulfonic acid groups that increase the polymer’s hydrophilicity and improve its stability in high-temperature and high-salinity environments. Additionally, the FPMs’ cross-linked structure enhances its resistance to thermal breakdown. This cross-linking provides mechanical integrity that helps the polymer maintain its structure and performance even under thermal stress.

Additionally, the AM-AMPS-Flu variant demonstrated favorable performance for enhanced oil recovery applications. This variant exhibited the best balance of thermal stability, swelling capacity, and fluorescence capabilities, making it particularly suitable for conformance control in high-temperature, heterogeneous reservoir conditions. The AM-AMPS-Flu microspheres maintained effective swelling behavior and structural integrity under challenging conditions while enabling real-time tracking through fluorescence. Based on these findings, we recommend the AM-AMPS-Flu FPM as the primary candidate for further field trials and optimization in enhanced oil recovery settings.

The incorporation of specific monomers within the FPM not only enhances its swelling behavior but also contributes to its thermal stability by forming a robust cross-linked network. This cross-linking improves mechanical integrity under thermal stress, effectively preventing premature degradation. Additionally, the presence of hydrophilic groups, such as amide and carboxyl functionalities, promotes strong interactions with water molecules, helping to mitigate thermal fluctuations and enhancing the microspheres’ resilience in extreme conditions. The findings emphasize the potential of FPMs to improve oil recovery processes in scenarios where conventional microspheres may prove ineffective.

It is important to consider the potential interactions between FPM and oil in heterogeneous reservoir environments. The presence of oil may influence FPM behavior through mechanisms such as solubility, adsorption, or potential chemical reactions with the polymer matrix. For instance, oil could impact the swelling capacity of FPM if there is partial solubility or adsorption of oil molecules onto the microsphere surface, potentially altering the polymer’s ability to absorb water and swell [51,52].

Moreover, the amphiphilic nature of some polymer components, like AMPS, could facilitate selective interactions with both water and oil phases, which might affect FPM’s distribution and retention within the reservoir. This interaction is critical, as it may alter the FPM’s effectiveness in blocking high-permeability pathways and enhancing conformance control in oil recovery applications.

While this study demonstrates the potential of FPMs as effective conformance control agents, several limitations should be considered. First, the laboratory-based synthesis and testing of FPM may not fully capture the complexities of reservoir conditions, such as extreme pressures, variable temperatures, and chemical interactions in diverse geological formations. Additionally, the long-term stability and degradation patterns of FPMs in high-salinity and high-temperature environments require further exploration, as these factors are critical for sustained performance in reservoir conformance control [52,53].

Future research should prioritize field-scale testing to better understand the in situ performance of FPMs under realistic reservoir conditions. These studies could help refine FPM formulations for enhanced durability and efficiency in high-salinity and high-temperature environments. Investigating the interaction of FPM with various reservoir rocks and fluids can also provide insight into optimizing their performance in different geological settings [54,55,56]. Finally, advancements in real-time monitoring technologies, such as advanced fluorescence detection systems, could improve our ability to track FPM movement and distribution within reservoirs, allowing for better assessment of conformance control and enabling more dynamic and responsive oil recovery processes.

## 3. Conclusions

The findings of this study highlight the potential of fluorescent polymer microspheres (FPM) as an innovative solution for reservoir conformance control. The key contributions of this research are as follows:

(1) The fluorescence properties of FPMs enable real-time tracking and improved reservoir management, offering a practical tool for monitoring fluid pathways;
(2) The significant swelling of FPMs in high-salinity and high-temperature conditions highlights their potential to effectively block high-permeability zones, improving fluid redirection and enhancing oil recovery efficiency;
(3) The incorporation of AMPS into the polymer matrix represents a significant advancement. It enhances the microspheres’ thermal stability, enabling them to withstand higher temperatures more effectively than conventional materials;
(4) The retention of superior swelling properties, despite the incorporation of fluorescent groups, underscores the robustness of FPM in brine environments. This, combined with enhanced thermal stability and real-time monitoring capabilities, establishes FPM as a highly advanced and efficient alternative to conventional polymers for applications in oil extraction.

This research marks a significant advancement by combining fluorescence with AMPS-enhanced polymer technology, offering a novel solution for conformance control and real-time monitoring.

## 4. Materials and Methods

### 4.1. Materials

Sorbitan Monostearate (Span 60), purchased from Shanghai Shanpu Chemical Co., Ltd. (Shanghai, China), was used as an oil phase dispersant. Anhydrous sodium carbonate (Na_2_CO_3_), supplied by Shanghai Hongguang Chemical Plant (Shanghai, China), was employed as an electrolyte. MBA (N,N′-Methylenebisacrylamide), chemically pure, supplied by Tianjin Guangfu Fine Chemical Industry Institute, was employed as a cross-linker. AMPS (2-acrylamido-2-methyl propanesulfonic acid), industrial grade, obtained from Shandong Quanxin Chemical Industry Co., Ltd. (Jinan, China), was used as a monomer. Ammonium persulfate (APS, used as water-soluble initiator), acrylamide (AM) monomer, fluorescein (Flu) fluorescent monomer, anhydrous ethanol, and other reagents used were all analytical-reagent grade, obtained from Sinopharm Chemicals Ltd. (Shanghai, China).

### 4.2. Synthesis and Purification of FPM

**Figure 8:**
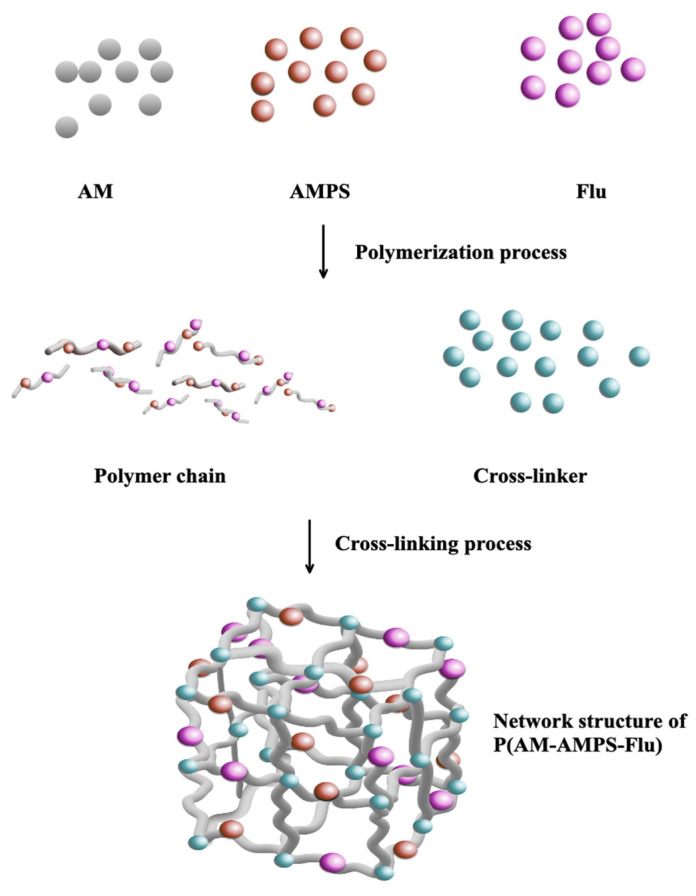
Schematic diagram of FPM synthesis.

**Figure 9:**
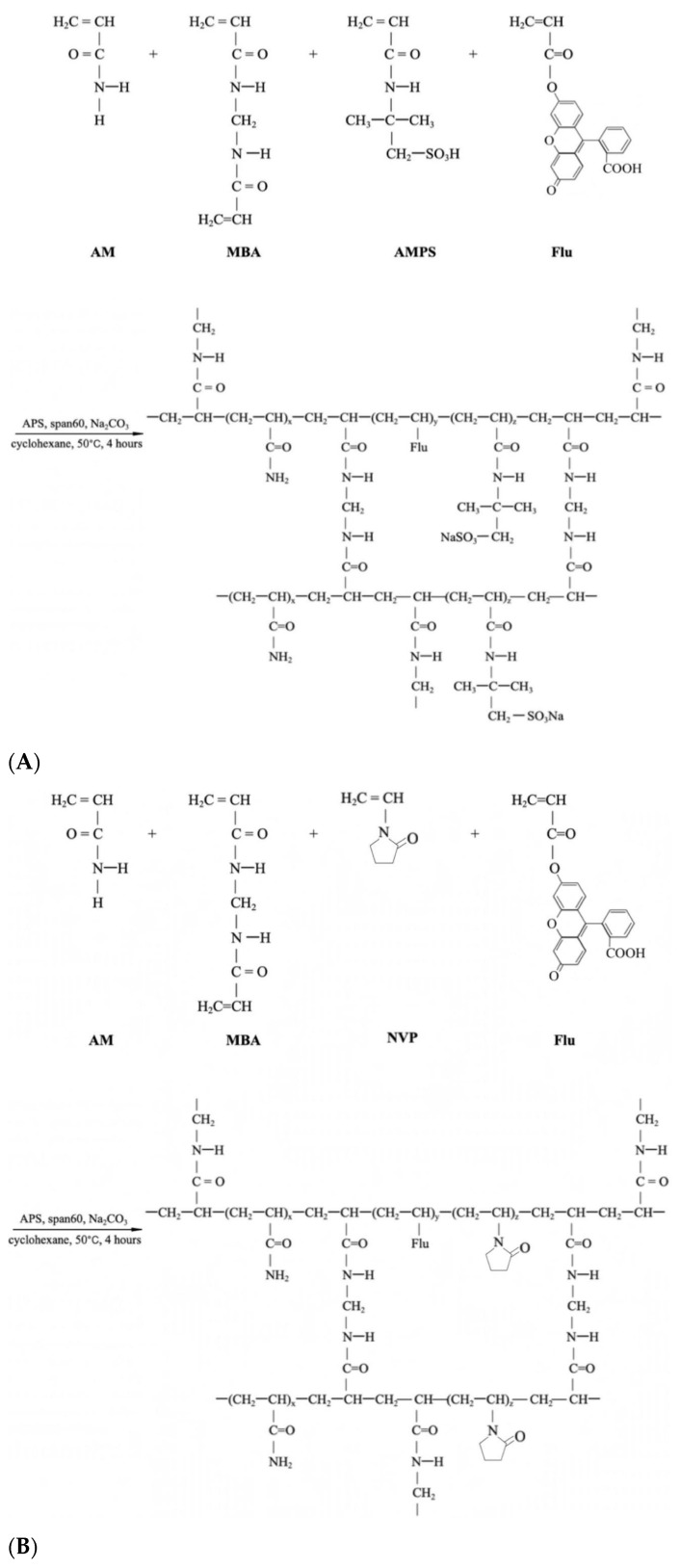

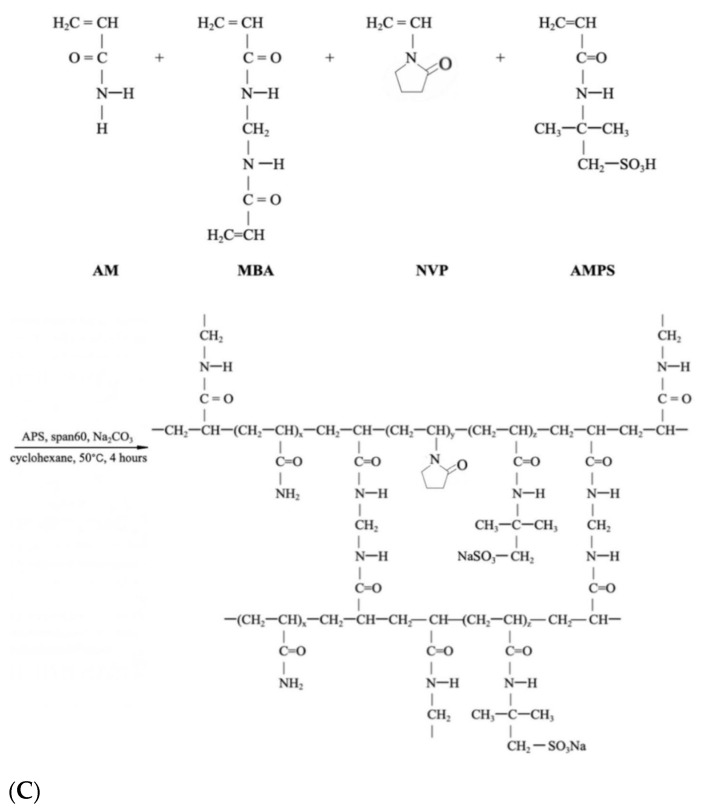
Synthesis scheme of (**A**). P(AM-AMPS-NVP), (**B**). P(AM-NVP-Flu), (**C**). P(AM-AMPS-NVP).

The preparation begins with a 300 mL four-neck flask equipped with a mechanical stirrer, reflux condenser, and a constant temperature water bath installation before the mentioned inverse suspension polymerization method was applied to prepare the FPM, where cyclohexane acted as the continuous phase and Span-60 as the nonionic polymeric surfactant in Figure 8 and Figure 9 [45].

The preparation of P(AM-AMPS-Flu) commenced by dissolving 2 g of AMPS, 10 g of AM, 0.52 g of MBA, and Na_2_CO_3_ in 21 mL of distilled water in Figure 9A. This solution was swiftly introduced into 60 mL of cyclohexane, previously purged with dry nitrogen, containing 0.005 g of Fluorescein and 4.8 g of Span 60 in the flask. A nitrogen-filled pipe was engaged to the flask with the oil phase in order to erase the air content. When aqueous droplets were observable within the oil solution, 4 mL of APS aqueous solution (75 g/L) was added to initiate the polymerization reaction [37]. The polymerization proceeded for 4 h at a temperature of 60 °C. At the specified time, FPM became apparent when stirring ceased. Subsequently, the precipitates were thoroughly washed with copious amounts of anhydrous ethanol and filtered using qualitative filter paper. The resultant product was then dried in a vacuum oven at 60 °C for 24 h. The previously described synthesis method was adapted to prepare different types of polymer microspheres by varying the monomer ratios. For P(AM-NVP-Flu) microspheres, we used a formulation of 10 g AM and 2 g NVP, shown in Figure 9B. For P(AM-AMPS-NVP) microspheres, the mixture included 8 g AM, 2 g AMPS, and 2 g NVP, which can be seen in Figure 9C.

### 4.3. Characterization Methods

FT-IR spectra of the FPM, the conventional polymer microsphere FPM, and the acrylamide monomer were analyzed using a Nicolet model NEXUS670 spectrometer (Thermo Fisher Scientific, Waltham, MA, USA), utilizing samples prepared on KBr pellets.

Observation of the surface morphology of these FPM was carried out employing a Leica DMI 3000B fluorescence microscope (Leica Microsystems, Wetzlar, Germany) and HITACHI SU8010 scanning electron microscopy (HITACHI, Tokyo, Japan). The average particle size of the FPM, both before and after swelling, was determined at room temperature using the RISE-2006 laser particle size analyzer provided by Jinan Runzhi Technology Co., Ltd. (Jinan, China). Fluorescence spectra of the FPM were collected using the Fluoromax-4 spectrometer (Horiba JobinYvon, Austin, TX, USA). To observe the structure of the FPMs after swelling, images were captured utilizing the FEI Quanta 200 FEG Environmental Scanning Electron Microscope (ESEM) by FEI Company, based in Holland. The microspheres’ thermal resistances were assessed using a SDT Q600 Simultaneous Thermal Analyzer (TA Instruments, New Castle, DE, USA), employing a heating rate of 10 °C/min up to 600 °C [35]. The microspheres, dispersed in water, were subjected to a high-temperature oven. Regular microscopic and Scanning Electron Microscope examinations were conducted at 24-h intervals to monitor the samples’ condition and changes.

## Figures and Tables

**Table 1:** Swelling behavior of microspheres with different monomers.

Polymer Microspheres	Initial Size (d)/μm	Size After 8 h Swelling/μm	Size After 24 h Swelling/μm	Size After 72 h Swelling/μm	Volumetric Swelling (D^3^/d^3^)
FPM	46	165	209	210	91
P(AM-NVP-Flu)	76	99	124	126	4
P(AM-AMPS-NVP)	55	100	139	139	15

## Data Availability

The original contributions presented in this study are included in the article. Further inquiries can be directed to the corresponding authors.
